# Feather corticosterone reveals that urban great tits experience lower corticosterone exposure than forest individuals during dominance-rank establishment

**DOI:** 10.1093/conphys/coad033

**Published:** 2023-05-27

**Authors:** Anders Brodin, Hannah Watson

**Affiliations:** Department of Biology, Naturvetarvägen 6A, Lund University, 223 62 Lund, Sweden; Department of Biology, Naturvetarvägen 6A, Lund University, 223 62 Lund, Sweden

**Keywords:** urbanization, intraspecific competition, corticosterone

## Abstract

Although the consequences of urbanization for the physiological health of animals are the focus of much active research, an overlooked aspect is how physiology could be indirectly modulated by the urban environment via changes in intraspecific behavioural interactions, particularly among gregarious species. Both urbanization and the establishment, as well as maintenance, of hierarchical rank position are processes that could incur physiological stress. Measurements of glucocorticoids (GCs) in relation to urbanization, however, have yielded inconsistent results. In most cases, GCs have been measured in blood, offering only a ‘snapshot’ of an animal’s current physiological state. Because circulating GCs are incorporated into growing feathers or hair, measurements of feather/hair GCs offer a longer term measure of stress exposure reflecting the whole period of feather/hair growth. During two calendar years, we collected tail feathers from 188 urban and forest great tits (*P. major*) across multiple sampling sites and analysed corticosterone (CORT—the main GC in birds) levels, reflecting CORT exposure during the extended period in late summer and early autumn when great tits moult and winter flocks are formed. Urban individuals exhibited consistently lower feather CORT (fCORT) levels than forest birds indicating lower overall exposure to CORT during this period. The lower fCORT levels in urban individuals could represent an adaptation to cope with the more challenging urban environment, physiological constraints on stress axis function or a trade-off between the ability to respond to stressors and predation risk during moult. Despite the expectation that CORT responses to urbanization are highly context-dependent, the spatial consistency of our results and agreement with a multi-population study of fCORT in European blackbirds (*Turdus merula*) suggests a generalization of the effect of urbanization on CORT exposure during post-breeding moult (*i.e.* not site- or species-specific).

## Teaser

Aggressive interactions between city-dwelling great tits could be more frequent due to higher population densities compared with forest habitats. However, urban great tits had lower levels of corticosterone (“stress hormones”) in feathers suggesting that competing for the top spots in a flock is less stressful in the city.

## Introduction

During the last 100 years, urbanization has changed landscapes dramatically, thereby changing species composition and richness (Blair, 1996; McKinney, 2008, 2002). Alongside climate change, urbanization represents one of the biggest challenges for global biodiversity (Hendry *et al.,* 2017). Organisms that live in urbanized habitats are faced with a variety of novel environmental cues, including changes in food sources, predators, microclimate, competing species, human disturbance and pollutant exposure (Sol *et al.,* 2011; Shanahan *et al.,* 2014; Papp *et al.,* 2015; Audet *et al.,* 2016; Cohen *et al.,* 2020), and species persistence likely requires a suite of behavioural and physiological adaptations to cope with this multitude of novel cues (Sepp *et al.,* 2017).

Although the effects of urbanization on wildlife have been the focus of intensive research in recent decades, an oft-overlooked aspect of city life is the possibility for changes in intraspecific interactions and competition due to the elevated densities that are often observed in urban populations (Hedblom and Söderström, 2012). For flock-forming birds, dominance rank is important for winter survival because higher ranked birds have priority access to food and hence will secure a more predictable winter food supply (Ficken *et al.,* 1990; Ekman and Lilliendahl, 1993; Krams *et al.,* 2010). Dominance-rank establishment in flocks is characterized by repeated aggressive interactions with conspecifics, with the most aggressive individuals securing the highest ranks (*e.g.*Hinde, 1952). Higher population densities (Hedblom and Söderström, 2012) and the presence of bolder individuals in urban environments (Senar *et al.,* 2017) could be expected to increase the frequency of aggressive interactions. Hence, the challenges faced by urban-dwelling animals may depend not only on characteristics of the urban habitat *per se* but also indirectly by effects of the urban environment on population densities.

In vertebrates, the regulation of the response to environmental stressors is mediated by the hypothalamic–pituitary–adrenal (HPA) axis and the associated secretion of glucocorticoid (GC) hormones (Sapolsky *et al.,* 2000; Romero, 2004). Although a GC stress response is clearly adaptive, by promoting phenotypic and behavioural changes that may increase survival, chronic stimulation of the HPA axis can have various negative effects. Although chronic stress is often thought to be associated with elevated levels of GCs, it is now understood that there is no consensus profile of a chronically stressed individual (Dickens and Romero, 2013). Dysregulation of the HPA axis can be manifest both in increases and decreases of GC levels, both of which can result in negative effects on health and performance (Boonstra, 2013). No doubt, this partly explains why measurements of stress hormones in vertebrates in relation to the degree of urbanization show no consistent pattern (reviewed by Iglesias-Carrasco *et al.,* 2020; Injaian *et al.,* 2020). Furthermore, as concluded in these two reviews, GC responses to urbanization are likely to be highly species-, population- and life stage-specific.

Another possible explanation for the lack of a consistent effect of urbanization on GC levels is that most studies have measured GCs circulating in the blood, which offer only a “snapshot” of an animal’s current state. An alternative is to measure GCs in feathers or hair, which offer a longer term measure because it reflects GC exposure over the whole period when feathers or hairs were growing (Bortolotti *et al.,* 2009). In birds, circulating corticosterone (CORT)—the major avian GC hormone—is passively deposited in growing feathers, thus reflecting an integrated measure of CORT exposure during the period of growth (Jenni-Eiermann *et al.,* 2015). However, it should be noted that CORT is not necessarily distributed evenly along the feather length, limiting the potential for relating feather CORT to events at specific time points during feather growth (Jenni-Eiermann *et al.,* 2015). Despite the clear advantages of measuring CORT in feathers (from hereon, fCORT), measurements in blood predominate, and relatively few studies have used feathers for quantifying CORT.

Studies in house sparrows (*Passer domesticus*) have shown similar fCORT levels between urban and rural adults (Hudin *et al.,* 2018). In contrast, urban juvenile house sparrows exhibited higher fCORT than rural juvenile sparrows, interpreted to indicate that young birds experienced developmental constraints in more urbanized environments (Beaugeard *et al.,* 2019). Another study showed that elevated metal concentrations were positively correlated with fCORT along an urbanization gradient in blackbirds (*Turdus merula*) (Meillère *et al.,* 2016), although, in the same species, rural blackbirds consistently had higher fCORT compared with urban blackbirds (Ibáñez-Álamo *et al.,* 2020).

The great tit (*Parus major*) is a flock-forming songbird that has successfully adapted to anthropogenic habitats such as residential gardens and city parks, whereas other populations remain in the original prime habitat of these species—the broadleaf deciduous forest. The great tit is a model organism in avian urban ecology, and there are numerous documented differences in demographic and phenotypic traits between great tits occupying urban and forest habitats. For example, urban great tits sing at higher frequencies (Slabbekoorn, 2013), have higher winter survival (Horak and Lebreton, 1998), have reduced reproductive success (Hõrak, 1993; de Satgé *et al.,* 2019), live at higher population densities (Solonen, 2001), exhibit different patterns of gene expression (Watson *et al.,* 2017) and DNA methylation (Watson *et al.,* 2021), show enhanced cognitive performance (Preiszner *et al.,* 2017) and have longer telomeres as adults (due to selective disappearance, Salmón *et al.,* 2017) compared with their non-urban dwelling counterparts. In late summer and early autumn, both adult and young great tits join large winter foraging flocks. The significance of establishing a high rank is demonstrated by the repeated aggressive interactions with conspecifics that are observed during this period (Hinde, 1952). This suggests that flock establishment is competitive and can be expected to increase exposure to CORT with ensuing negative effects (Breuner *et al.,* 2008). Elevated population densities in urban and suburban areas have been attributed to winter feeding by humans and provision of artificial nesting cavities, both of which could also directly influence intraspecific competition during the period of flock formation.

The aim of the present study was to investigate whether urban and forest great tits differ in their exposure to CORT during the period of flock formation and dominance rank establishment in late summer and early autumn. This period of flock formation coincides with feather moult between July and October, during which adults undergo complete moult, whereas juveniles exhibit a partial moult, meaning that both age categories moult their tail feathers during this period. The full growth of a tail feather in a bird such as a great tit takes around a month (Brodin and Ekman, 1994); therefore, fCORT in a fully grown tail feather will reflect an integrated measure of CORT exposure during this time window. Although this presents a limitation for investigating CORT exposure outside of the moult period (Romero and Fairhurst, 2016), the time window of fCORT deposition is ideal for our question concerning the period of flock formation.

We set out to test two competing hypotheses. One hypothesis is that higher population densities will increase the frequency of aggressive interactions with conspecifics and subsequent stimulation of the HPA axis and CORT secretion, resulting in higher fCORT in urban birds compared with forest birds. An alternative hypothesis is that urban habitats offer an easier foraging environment (*e.g.*Horak and Lebreton, 1998; Schoech *et al.,* 2007), which could lead to relaxed competitive interactions, reduced stimulation of the HPA axis and lower fCORT in urban, compared with forest, birds.

## Methods

### Field data collection

We captured 188 great tits (adults: *n* = 158; juveniles: *n* = 30) from winter 2018–2019 to winter 2019–2020 at seven urban and seven forest sites. Birds were captured in mist nets during winter or in nestboxes during spring. Urban sites comprised urban parkland and residential areas in the cities of Malmö (lat. 55.59, long. 13.01, population: 348 000) and Lund (lat. 55.42, long. 13.20, population: 126 000), both located in southernmost Sweden. Forest sites were located in areas of low human habitation (<5 inhabitants/km^2^) and dominated by deciduous forest. Six forest sites were located in southern Sweden, and one forest site was located in central Sweden. The sites in southern Sweden were dominated by broadleaf tree species such as common oak (*Quercus robur*), common beech (*Fagus sylvatica*), Scots elm (*Ulmus glabra*), European ash (*Fraxinus exelcior*) and birch (*Betula spp*). The forest site in central Sweden was covered by a mixed coniferous/deciduous forest with Scots pine (*Pinus sylverstris*), Norwegian spruce (*Picea abies*), birch (*Betula spp*.) and trembling aspen (*Populus tremula*). Great tits rarely disperse >1 km (Verhulst *et al.,* 1997) and maintain a typical winter home range of 0.05 km^2^ (Krams *et al.,* 2006); therefore, exchange between urban and forest populations is unlikely.

In most cases, two feathers (both second outermost rectrices) were collected from each bird. We did not collect rectrices undergoing moult or rectrices from first-year birds that had not been moulted. In a few cases, only one rectrix could be collected. It was assumed that birds had moulted their tail feathers during the preceding autumn and flock formation. Although it is possible that some birds could have lost and regrown tail feathers after this period, we would expect this to occur at random among sexes and habitats. Earlier onset of moult has been reported in urban house sparrows (Hudin *et al.,* 2018) and urban chickadees (Hope *et al.,* 2016), which is likely due to earlier onset of breeding, given that timing of moult is highly correlated with the end of breeding (Siikamaki *et al.,* 1994). Because we typically observe breeding, on average, 1 day earlier in our urban populations (H. Watson, unpubl. data), we do not expect to see any biologically meaningful difference in timing of moult. We recorded sex, age (adult or juvenile, based on flight feather moult pattern) and body mass. Birds were marked with a uniquely numbered metal ring and released after processing.

### CORT extraction and quantification

Feathers were measured with calipers from the base to the tip of the vane to the nearest 0.1 mm, and mass was recorded to the nearest milligram. CORT was extracted from whole intact feathers according to a protocol for ‘cold’ extraction (Will *et al.,* 2019). After removal of the calamus, two tail feathers (or in some cases, one, where a bird did not possess two second outermost rectrices) per bird were placed in a glass tube with 9 ml methanol (ensuring feather was fully immersed) and incubated at 4°C for 20 h. After cold incubation, feathers were discarded, and tubes were placed in a heating block at 50°C, while methanol was evaporated under a gentle stream of nitrogen gas. Samples were resuspended in 250 μl assay buffer (provided in kit—see below) and stored at −20°C until quantification. For each round of extractions, a methanol blank was run alongside the samples (mean ± SD: 2.39 ± 0.74 pg·ml^−1^). fCORT was quantified by ELISA assay (Cayman Chemical, MI, USA, product no. 501320) according to the manufacturer instructions. Samples were run in duplicate, alongside a standard curve ranging from 8.2 to 5000 pg·ml^−1^ also run in duplicate on each plate. fCORT concentrations were calculated according to the standard curve, and all samples were at least one standard deviation from the mean methanol blank (taken to be the detection level). Inter- and intra-assay variation (mean ± SE) were 3.36 ± 0.44% and 8.95 ± 1.78%, respectively.

### Statistical analysis

We carried out the statistical analysis in R 4.1.0 (R Core Team, 2021). We expressed fCORT values relative to feather length as picograms per millimetre of feather. A general linear mixed model was fitted to log-transformed values of fCORT using the lmer package. The maximal model included the fixed effects of habitat (forest/urban), sex (female/male), age (juvenile/adult), body mass (g), year (2018/2019), total feather mass (mg), the interactions of habitat:age, habitat:sex and age:sex and the random effects of site and assay number. No birds were measured twice. Although we corrected fCORT values for feather length, we deemed it necessary to additionally control for feather mass (by including feather mass as a covariate) because detection of fCORT is known to be inflated in small samples (Berk *et al*., 2016, Lattin *et al.,* 2011). We performed a stepwise backwards elimination to arrive at a minimum adequate model. Terms were eliminated if they did not significantly improve the model fit, as assessed by *F*-tests between nested models. *P*-values and degrees of freedom for significant parameters were estimated using *F*-tests with the Satterthwaite method. We performed *post hoc* tests on significant interactions using the emmeans package and *P*-value correction with the Tukey method. Data are presented for 113 forest birds (females = 54, males = 59) and 75 urban birds (females = 45, males = 30).

## Results

**Figure 1:**
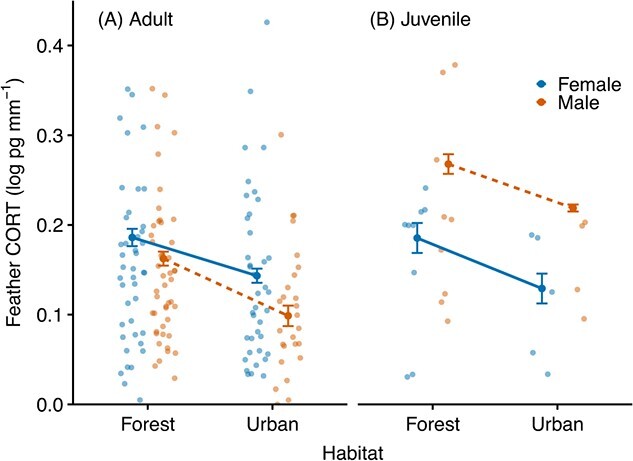
Concentration of corticosterone in feathers (fCORT) of forest- and urban-dwelling great tits, in (A) adults (*n* = 158; 84 females, 74 males) and (B) juveniles (*n* = 30; 15 females, 15 males), illustrating females (blue, solid lines) and males (orange, dashed lines) and reflecting CORT exposure during the period of feather moult. Mean ± SE of fitted values from the minimum-adequate model are shown with raw data values. Note that six raw values are omitted from the plot in the range of 0.55–0.85 pg·mm^−1^ to facilitate interpretation.

fCORT was significantly lower in urban birds compared with those living in forest environments (Fig. 1; *ß*_urban_ = −6.08^e-2^ ± 2.12^e-2^, *F*_1,9.7_ = 8.2, *P* = 0.017). On average, urban birds had fCORT levels of 0.11 ± 0.07 pg·mm^−1^ lower than forest birds. Although a significant interaction suggested fCORT significantly differed between sexes in an age-dependent manner (*F*_1,176.2_ = 4.4, *P* = 0.038), *post hoc* tests revealed no significant pairwise contrasts (all *P* > 0.1). The biggest difference was among males, with juvenile males tending to have, on average, 0.15 ± 0.08 pg·mm^−1^ higher fCORT than adult males (Fig. 1; contrast: *t* = −2.21, *P* = 0.12). fCORT was also negatively correlated with the amount of feather used, as indicated by total feather mass (*ß* = −9.21^e-3^ ± 3.67^e-3^, *F*_1,79.7_ = 6.3, *P* = 0.014). fCORT did not vary with year or body mass (all *P* > 0.4). The effect of habitat was consistent across both sexes (*ß*_urban:male_ = −6.16^e-3^ ± 4.22^e-2^, *F*_1,114.1_ = 2.1^e-2^, *P* = 0.88) and age groups (*ß*_urban:juvenile_ = −2.94^e-2^ ± 5.45^e-2^, *F*_1,116.5_ = 0.29, *P* = 0.59). The variance associated with the random effects in the minimum adequate model was 5.37^e-4^ ± 2.32^e-2^ and 3.32^e-3^ ± 5.77^e-2^ for site and assay number, respectively.

## Discussion

We found significantly lower levels of fCORT in urban-dwelling birds compared with forest birds, indicating that urban birds did not experience elevated CORT exposure during the period of moult and flock formation, as might have been expected if higher densities resulted in increased interspecific interactions and/or the urban environment induces elevated adrenocortical activity. Replication of measurements across multiple urban and forest sites suggests that the lower CORT exposure of urban great tits is a general pattern and can be attributed to the urban environment *per se*. Although it is frequently assumed that coping with anthropogenic habitats is cognitively demanding (Sol *et al.,* 2011; Papp *et al.,* 2015; Audet *et al.,* 2016) and challenging in general (Atwell *et al.,* 2012; Bailly *et al.,* 2016), other pressures may be ameliorated through the provision of food and artificial nesting sites by humans (Amrhein, 2014). Although physiological stress can be manifest by both increases and decreases in CORT exposure, our observations suggest that urban great tits are not adversely affected by the urban environment because they avoid the damaging effects of elevated CORT exposure. Lower CORT exposure of urban birds could arise via reduced baseline levels and/or dampened stress responses, both of which could be adaptations to avoid the negative effects associated with overstimulation of the HPA axis when living in a ‘stressful’ environment (McEwen and Wingfield, 2003). Environmental stimuli in the urban environment, such as noise, artificial light and human disturbance could all be sources of overstimulation of the HPA axis.

There is some evidence that the urban environment selects for species and individuals with reduced adrenocortical responses to novel stressful events. Atwell *et al.* (2012) found that urban environments select for reduced CORT responses in conjunction with increased boldness. In a common garden experiment, dark-eyed juncos (*Junco hyemalis*) that had recently colonized a novel urban environment had undergone rapid adaptive evolution of traits that are considered to be beneficial in urban environments. Urban juncos displayed attenuated plasma CORT responses during a standardized stress test and displayed bolder exploratory behaviour than their rural conspecifics, which was suggested to be driven by habituation to frequent stressors in the urban environment. Habituation, expressed as dampened adrenocortical responses, has also been observed in Magellanic penguins (*Spheniscus magellanicus*) in response to human disturbance in various settings (Walker *et al.,* 2006).

Habituation is thus a viable explanation for the observed reduced CORT exposure of urban great tits. Selection for reduced GC responses in urban-dwelling great tits may also be accompanied by increased boldness, as observed in the juncos (Atwell *et al.,* 2012). Indeed, research has repeatedly found urban great tits to be ‘bolder’ than their forest-dwelling counterparts (Senar *et al.,* 2017; Caizergues *et al.,* 2022). Senar *et al.* (2017) found that urban great tits were more ‘aggressive’, eliciting more fear screams and pecking more than forest birds when handled. These behaviours are consistent with a ‘proactive’ coping style, which is typically associated with reduced adrenocortical stress responses (Koolhaas *et al.,* 1999). Nonetheless, another study found urban great tits (along with 14 other passerine species) were less aggressive, yet at the same time emitted more fear screams, suggesting complex covariation between these behavioural traits (Møller and Ibáñez-Álamo, 2012). A study of house sparrows (Vincze *et al.,* 2016) showed that urban birds were faster to habituate to urban stressors than rural birds, in which case urban environments may not necessarily be predicted to affect CORT levels in any specific direction. Consistent with this, many studies have failed to find consistent associations between behavioural traits and adrenocortical responses (Réale *et al.,* 2010), and it is now generally considered that the relationship between behaviour and hormones is more complicated than the dichotomy of early models of coping styles (Koolhaas *et al.,* 2010).

An alternative explanation for the observed lower fCORT levels in urban birds is that the urban environment is not as unfavourable for birds, such as the great tit, as often thought. Schoech *et al.* (2007) showed that suburban Florida scrub jays (*Aphelocoma coerulescens*), fed by human visitors, were tamer and had lower plasma baseline CORT levels than wildland birds. By providing wildland scrub jays with supplemental food, the authors showed that CORT decreased to the same levels as in suburban birds. The link between food availability and CORT is well established in birds, whereby increased availability of food leads to reduced circulating CORT levels (*e.g.*Kitaysky *et al.,* 2001). In Sweden, the great tit is the most common visitor to bird feeders in winter. Although bird feeding, in Sweden and elsewhere in Europe, does not usually start until after great tits moult their tail feathers, great tits are ‘experts’ in obtaining food from anthropogenic sources (Johnsson and Brodin, 2019; Brodin, 2020), and it is possible that the availability of anthropogenic food sources is higher in urban habitats already in late summer/early autumn. If urban great tits experience more abundant food and reduced competition for food during moult and flock formation, this could explain the observed lower CORT exposure compared with forest great tits during this life history stage.

Another possibility is that there could be differences in the seasonal regulation of CORT between urban and forest birds. It has been widely found that passerine birds downregulate CORT—both basal and higher circulating levels—during the period of moult (Romero, 2004), and experimental studies have shown that CORT reduces rate of feather regrowth (Romero *et al.,* 2005) and feather quality (Lattin *et al.,* 2011). The ability to complete moult rapidly would thus seem to be of great importance. If predation risk is higher in urban sites, this could select for enhanced CORT suppression and more rapid moult in urban birds compared with forest birds. However, it is unclear how predation risk differs between urban and forest sites, and it is probably highly site- and species-specific. Although evidence suggested that urban house sparrows experienced higher predation risk (Seress *et al.,* 2011), other studies suggest birds are released from predation pressure in urban environments (Gering and Blair, 1999; Møller and Ibáñez-Álamo, 2012).

A yet further possibility is that the observed differences in CORT exposure between urban and forest great tits are explained by variation in metabolic rate. Jimeno *et al.* (2018) showed that differences in CORT levels reflected variation in metabolic rate rather than stress (either psychological or physiological). Because GCs induce gluconeogenesis, a high metabolic rate could be expected to be consistent with higher circulating levels of GCs to meet high metabolic demands. However, there are no clear expectations for habitat-driven differences in metabolic rate during the period of moult and flock formation, when food is generally abundant and temperatures mild. Although the faster pace of life commonly attributed to urban great tits (Senar *et al.,* 2017; Caizergues *et al.,* 2022) would be expected to be consistent with a higher metabolic rate, one study (from one of our urban sampling sites) found urban great tits had a consistently lower metabolic rate than their forest conspecifics (Andersson *et al.,* 2018).

Even if we cannot be sure of the causal mechanism underlying the observed habitat differences in fCORT, lower fCORT in urban great tits demonstrates that they do not experience increased CORT exposure due to the pressures imposed by the urban environment *per se* or increased interspecific interactions during the critical period of moult and flock formation. The replicability of the results across sampling sites suggests this is a general pattern, at least in the study species. In a multi-population study of ten European populations of blackbirds, Ibáñez-Álamo *et al.* (2020) also found that urban birds consistently had lower levels of fCORT than rural populations and, supported by other measures of physiological stress, concluded that the urban environment was not detrimental for this species. Lower CORT exposure in both the urban blackbirds (latter study) and great tits (present study) could indicate an easier foraging environment, consistent with the finding of lower plasma baseline CORT in suburban Florida scrub jays that have access to *ad libitum* human-provided food year-round (Schoech *et al.,* 2004).

In conclusion, urban great tits had lower levels of CORT in feathers that had grown in late summer and early autumn, indicating a lower overall exposure to CORT during this period, coinciding with the period of flock formation and associated high interspecific interactions. It is unclear whether reduced CORT secretion in urban birds represents an adaptation to city living, physiological constraints imposed by the urban environment or that the urban environment is not as unfavourable for great tits as often thought. It remains to be determined if CORT exposure is lower in urban birds throughout the rest of the annual cycle or only during the period of moult. To derive long-term CORT measures outside of the moult period is more challenging. It would be necessary to capture great tits throughout the year and remove two tail feathers, forcing birds to grow new feathers; these individuals would then need to be recaptured to collect the newly grown feathers for quantification of CORT. The trend for higher fCORT in juvenile male great tits, compared with adults, is also worthy of further investigation. Regardless of the underlying mechanism(s), our observation of significant differences between urban and forest great tits, replicated across sampling sites, demonstrates differences in the physiological response to their respective environments during the establishment of dominance hierarchies.

## Funding

This work was supported by the Carl Tryggers Foundation (grants CTS 18:51 and 21:1394 to A.B.).

## Data availability statement

Data available from the Figshare data repository https://doi.org/10.6084/m9.figshare.22698589 (Watson and Brodin, 2023).

## Author contributions

A.B. conceived the study and carried out most of the fieldwork with some support from H.W. Laboratory and data analyses were performed by H.W. A.B. and H.W. jointly wrote the manuscript.
